# Enacted Sexual Minority Stigma, Psychological Distress, and Sexual and Drug Risk Behaviors Among Urban Men Who Have Sex with Men (MSM)

**DOI:** 10.1007/s10461-022-03784-5

**Published:** 2022-07-13

**Authors:** Francesca Silvestri, Carla Tilchin, Jessica Wagner, Matthew M. Hamill, Anne Rompalo, Khalil G. Ghanem, Christina Schumacher, Sebastian Ruhs, Adena Greenbaum, Carl Latkin, Jacky M. Jennings

**Affiliations:** 1 Center for Child and Community Health Research (CCHR), Department of Pediatrics, Johns Hopkins School of Medicine, Baltimore City, MD USA; 2 Department of Mental Health, Johns Hopkins Bloomberg School of Public Health, Baltimore City, MD USA; 3 Department of Infectious Disease, Johns Hopkins School of Medicine, Baltimore City, MD USA; 4 STI/HIV Program, Baltimore City Health Department, Baltimore City, MD USA; 5 Chase Brexton, Baltimore City, MD USA; 6 Department of Health, Behavior and Society, Johns Hopkins Bloomberg School of Public Health, Baltimore City, MD USA

**Keywords:** MSM, Stigma, Transactional sex, Mental health, STI control, HSH, Estigma, Sexo transaccional, Salud mental, Control de las ITS

## Abstract

Urban Black men who have sex with men (MSM) bear a disproportionate burden of HIV and syphilis in the U.S. Experiences of enacted sexual minority stigma and psychological distress among these men may be associated with HIV/STI sexual and drug risk behaviors. The objective was to determine the associations between enacted sexual minority stigma, psychological distress, and sexual and drug risk behaviors. In an urban prospective cohort study, survey measures assessed past 3-month exposure to enacted sexual minority stigma, psychological distress, and sexual and drug risk behaviors. Multivariable logistic regression models were utilized for hypothesis testing. The Black MSM (N = 140) reported the following: 22.1% experiences of enacted sexual minority stigma, 39% high levels of psychological distress, 48.6% > 1 sex partner, 8.6% transactional sex, and 6% injection drug use (IDU). In models adjusted for age and education, enacted sexual minority stigma significantly increased the odds of reporting > 1 sex partner, transactional sex, and IDU. Adjusting additionally for homelessness, the association between enacted sexual minority stigma and transactional sex remained significant. Adding psychological distress to this model showed a significant association between psychological distress and transactional sex, while the association was no longer significant for transactional sex. These findings highlight some of the complex psycho-social relationships that may be associated with sexual and drug risk behaviors among Black MSM placing them at increased risk for HIV and syphilis.

## Resumen

Hombres urbanos de raza Negra que tienen sexo con hombres (HSH) sobrellevan una carga desproporcionada de VIH y sífilis en los EE.UU. Experiencias de estigma efectivo de minoría sexual y angustia psicológica entre estos hombres pudiese ser asociado con conductas sexuales de riesgo VIH/ITS y drogas. El objetivo era determinar las asociaciones entre un estigma efectivo de minoría sexual, angustia psicológica, y comportamientos sexuales y de riesgo de drogas. En un estudio de cohortes prospectivo urbano, las medidas de la encuesta evaluada en los últimos tres meses de exposición al estigma efectivo, angustia psicológica, y sus conductas sexuales y comportamientos riesgoso de drogas. Modelos de regresión logística multivariante se utilizaron para la prueba de hipótesis. Los HSH de raza negra (N = 140) reportaron lo siguiente: 22.1% experiencias de estigma efectivo, 39% niveles altos de angustia psicológica, 48.6% y > 1 pareja sexual, 8.6% sexo transaccional, y 6% uso de drogas inyectables (UDI). En modelos ajustados a edad y educación, un estigma efectivo de minoría sexual aumentó de manera significante las probabilidades de reportar y > 1 pareja sexual, sexo transaccional, y UDI. Ajustando adicionalmente para personas sin vivienda, la asociación entre estigma efectivo de minoría sexual y sexo transaccional permaneció significante. La adición de angustia psicológica al modelo mostró una asociación significativa entre angustia psicológica y sexo transaccional, mientras que la asociación ya no era significativa para el sexo transaccional. Estos resultados destacan algunas de las complejas relaciones psicosociales que pudiesen estar asociadas con conductas sexuales y de riesgo de drogas entre HSH de raza negra, poniéndolos a mayor riesgo de contraer VIH y sífilis.

## Introduction

Urban Black men who have sex with men (MSM) and specifically, Black MSM experience the highest rates of HIV and syphilis in the United States (U.S.). In 2019, Black MSM accounted for 37.9% of new HIV diagnoses in the U.S., a proportion greater than any other racial/ethnic group [1]. In 2018, 54% (18,760) of all primary and secondary (P&S) syphilis cases were among MSM [2] and co-infection with HIV is high; 42% of MSM P&S cases were also living with HIV [3].

While studies on MSM who engage in transactional sex, which can be defined as “the commodification of the body in exchange for shelter, food, and other goods and needs” [4] are few, existing data suggests that MSM reporting transactional sex have higher rates of STIs and HIV compared to other MSM [5, 6]. Previous work, for example, has found that MSM who report frequent transactional sex (i.e. 11 or 12 times in the past 12 months) had a higher odds of an HIV diagnosis [7]. Prevalence estimates of transactional sex among MSM range from 16 to 29% among industrialized countries [4, 8, 9]. One study conducted from 2012 to 2015 in Baltimore City among adult Black MSM found that 29% of men reported transactional sex in the past 90 days [4]. The variability in prevalence estimates may be due in part to differences in the definitions of transactional sex and issues of under-reporting as well as different ways through which MSM become involved in transactional sex.

One factor that has been shown to be associated with transactional sex and other HIV/STI sexual and drug risk behaviors among MSM has been reported experiences of enacted sexual minority stigma related to sexual orientation. Enacted sexual minority stigma is defined as overt, negative actions of verbal harassment, discrimination, and or physical assault directed at an individual because of their sexual orientation, and is often termed homophobia [10, 11]. In 2015, the American Men’s Internet Survey (AIMS), a cross-sectional study among MSM in the U.S., found that the most reported lifetime stigma experiences included verbal harassment (57%), being subject to family gossip (50%) and feeling scared to be in public places because of same-sex sexual behavior (32%) [12]. In the 2011 National HIV Behavioral Surveillance System (NHBS), experience of enacted sexual minority stigma was reported by 24% of MSM respondents, and specific types of enacted sexual minority stigma including verbal harassment, discrimination, and physical assault separately were found to be associated with a 41%, 49%, and 72% increase in the prevalence of transactional sex among MSM, respectively [13, 14]. In a meta-analysis of studies from 1992 to 2017, MSM who experienced homophobia were at significantly increased odds of reporting any sexual risk behavior (OR 1.33, 95% CI 1.25, 1.42) including an increased number of sex partners (OR 1.16, 95% CI 1.13, 1.19) with effect sizes larger for samples with ≥ 50% Black (vs. White) MSM [15].

**Fig. 1:**
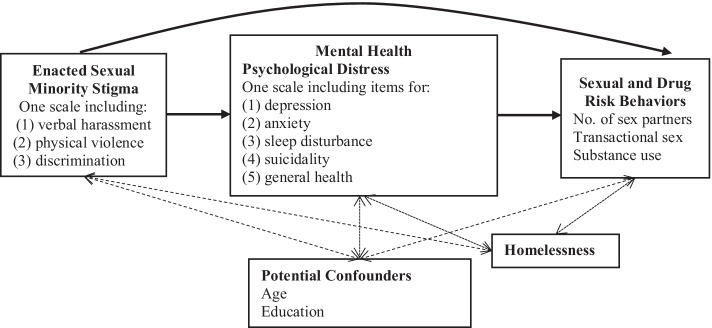
Conceptual framework depicting the hypothesized relationships between enacted sexual minority stigma, psychological distress and sexual and drug risk behaviors and including potential confounders and homelessness

Enacted sexual minority stigma has been shown to increase psychological distress including depression, anxiety, sleep disturbance, suicidality and poor general health [10, 16]. One hypothesis is that increases in psychological distress due to experiences of enacted sexual minority stigma may result in coping behaviors which place an individual at greater risk for STIs and HIV [10, 16, 17]. These coping behaviors may include, for example, increases in sexual and drug risk behaviors such as increased numbers of sex partners, transactional sex, and substance use [18–22]. The hypothesized relationships between enacted sexual minority stigma, psychological distress and sexual and drug risk behaviors are depicted in Fig. 1.

Prior research, however, has been limited in several ways. Prior research on enacted sexual minority stigma among MSM, such as in the NHBS and other national surveillance reports, has not included an analysis among a racial subgroup on experiences of enacted sexual minority stigma, which is a critical relationship to explore. Additionally, there is limited evidence exploring the role of psychological distress in the hypothesized link between enacted sexual minority stigma and transactional sex as well as other sexual and drug risk behaviors among urban Black MSM. Previous work among young Black MSM demonstrated an association between stigma and psychological distress, but enacted sexual minority stigma was not investigated specifically [21]. A closer look at this pathway has important implications in contextualizing racial/ethnic disparities of HIV and syphilis acquisition and transmission, particularly in areas where there is a high prevalence of transactional sex among MSM, such as in Baltimore City, Maryland [18]. Potentially complicating these links are additional intersecting issues such as homelessness which may be prevalent among Black MSM [20].

The objectives of these analyses were among a cohort of Black MSM to determine the association between (1) enacted sexual minority stigma and sexual and drug risk behaviors, and (2) enacted sexual minority stigma and psychological distress. In addition, we sought to determine the role of psychological distress in any significant relationship between enacted sexual minority stigma and a sexual and drug risk behavior. Black MSM were the focus of this study given the disparities in STIs/HIV among these men and the advantage that the majority of the cohort self-identified as Black.

## Methods

### Overview

The data for this study came from the Understanding Sexual Health in Networks (USHINE) study, a prospective cohort study conducted by the Johns Hopkins Center for Child and Community Health Research (CCHR) in collaboration with the Baltimore City Health Department (BCHD) and the Centers for Disease Control and Prevention (CDC). The overall goal of USHINE is to understand the network epidemiology of syphilis among MSM to identify ways to augment and strengthen local health department practices regarding the syphilis prevention-care continuum. Study recruitment and follow-up sites include three clinical sites (two public sexual health clinics, one primary care clinic) and one community-based organization. Participants were eligible if they were 18 to 45 years of age, male, were a resident of Baltimore City, reported sex with a male in the past 6 months, and spoke English. Study visits included an audio-computer self-assisted (ACASI), behavioral survey at baseline and every 3 months. Data were utilized from the 9-month follow-up survey which included the enacted sexual minority stigma and psychological distress measures and was collected from September 2019 to October 2020. The current analysis was restricted to Black participants only as a main objective of the analysis given the higher burden of HIV and syphilis infection in this population and the unique experiences Black MSM face.

### Measures

The survey assessed individuals’ demographics (age, education, employment), and homelessness in the past 3 months. Experiences of homelessness were measured with the following question “In the past 3 months, have you been homeless? By homeless, I mean you were living on the street, in a shelter, in a single room occupancy (SRO) hotel, with friends, in a car, or you haven’t had a regular place to stay for at least one night in the past 3 months?” To measure enacted sexual minority stigma, the primary exposure of interest, questions were adapted from the NHBS [13] which were derived from previous work among MSM on stigma, harassment, and discrimination [23–27]. Participants were asked if in the past 3 months they had experienced any of the following situations because someone knew or made assumptions about their sexuality: (1) verbal harassment, measured by whether the participant had been called names or verbally insulted, (2) discrimination, measured by whether the participant received poorer services, had been treated unfairly at work or school, or had been denied or given lower quality healthcare by someone, and (3) physical violence, measured by whether the participant had been physically attacked or injured. The three enacted sexual minority stigma measures were assessed with as experienced or not experienced in the past 3 months. The three measures were summed and any value greater than one was classified as enacted sexual minority stigma compared to no report.

Psychological distress, considered a potential factor on the pathway between enacted sexual minority stigma and transactional sex in this analysis, was measured with questions adapted from Diaz and colleagues’ work on assessing sexual risk as an outcome of social oppression, which encompasses experiences of homophobia, racism, and poverty [14]. These measures were validated among minority MSM in three large U.S. cities. The prevalence of the following psychological symptoms in the past 3 months were measured: [1] depression, measured by how often the participant felt sad or depressed, [2] anxiety, measured by how often the participant felt scared or panicky for no apparent reason, [3] sleep disturbances, measured by how often the participant had difficulty sleeping, [4] suicidality, measured by how often the participant felt like taking their own life, and [5] general health, measured by how often the participant felt sick, ill, or not well. All psychological distress measures were assessed with a 5-item scale using a 4-point frequency scale ranging from never, once or twice, a few times, to many times. All items were summed to create an ordinal cumulative score measuring psychological distress.

The outcomes of interest included sexual and drug risk behaviors. Sexual behaviors measured included number of sex partners in the past 3 months (dichotomized as 0–1, > 1), unprotected anal intercourse (UAI) at last sex, and transactional sex. To measure transactional sex, the participant was asked in two questions (yes/no) if they gave and/or separately, received sex for money, drugs, or something else in the past 3 months. If the participant responded yes to either giving or receiving, they were considered as having engaged in transactional sex in the past 3 months. Drug behaviors included injection drug use (IDU) and non-IDU substance use. IDU was measured by the question, “In the past 3 months, have you shot up or injected drugs that were NOT prescribed for you?” Non-IDU was measured by a question “In the past 3 months, have you used any non-injection drugs (drugs you did not inject) other than those prescribed for you?” In addition, participants were asked if they utilized any injection or non-injection drugs prior or during sex in the past 3 months.

### Statistical Analysis

In exploratory analysis, statistical testing (chi-square and t-tests, where appropriate) was conducted to test for associations between the baseline characteristics of Black men included vs. not included in the analytic sample. Following, statistical testing was conducted to examine associations between enacted sexual minority stigma, baseline demographic characteristics, sexual and drug risk behaviors (outcomes objective one), and psychological distress (outcome objective 2). While age and education were not significantly associated with any outcome in bivariate analyses, these were adjusted for in final multivariable analyses due to potential confounding [13, 18, 20]. Unadjusted and adjusted logistic regression models were utilized for hypothesis testing of the sexual and drug behavior outcomes. In the adjusted models, we tested the direct relationship between enacted sexual minority stigma and each outcome controlling for age and education, and in a separate series of models, we also controlled for homelessness. In a final model, we tested the direct relationship between enacted sexual minority stigma and transactional sex while including psychological distress in the model and controlling for age, education, and homelessness. All statistical analyses were performed using Stata Version 17.0. Statistical significance was determined by a confidence interval that did not cross 1.0 and a p-value of less than 0.05.

## Results

At baseline, 408 MSM enrolled in the study and 73.0% self-identified as Black. Forty-seven percent of enrolled MSM completed the 9-month survey, among whom 73.7% were Black MSM and were included in this analysis. Baseline characteristics including demographics, the main exposure (i.e. enacted sexual minority stigma) and each of the three outcomes of Black men included in the analytic sample (N = 140) compared to those not included (N = 163) were not significantly different (*data not shown)*.

In the analytic sample, the average age of participants was 31.0 [standard deviation (SD) 6.1] years (Table 1) and most of the participants, 32.1%, were 25–29 years of age (*data not shown)*. Forty-eight percent had less than or equal to a high school education, and 37.1% reported that they were currently not working. Nineteen percent had experienced homelessness in the past 3 months, 48.6% reported greater than one (> 1) sex partner in the past 3 months, 57.1% reported UAI in the past 3 months and 8.6% reported transactional sex in the past 3 months. Six percent reported IDU and 20.0% reported non-IDU substance use in the past 3 months. The mean score of psychological distress was 4.8 (SD 3.6). Thirty-nine percent of participants reported high (greater than the mean) levels of psychological distress, and 22% reported one or more types of enacted sexual minority stigma, including 17.9% reporting verbal harassment, 6.4% reporting physical violence, and 12.9% reporting discrimination in the past 3 months (*data not shown in table)*.

**Table 1:** Participant characteristics by enacted stigma^a^ among Black gay and bisexual men (MSM) participating in the 9-month study visit of the USHINE study, Baltimore City, 2019- 2020 (N = 140)

Characteristics	Overall N = 140	Enacted stigma^a^ n = 31 (22.1)	No enacted stigma^a^ n = 109 (77.9)	Test statistic^d^
	Mean (SD)	Mean (SD)	Mean (SD)	
Demographics				
Age, years	31.0 (6.1)	29.6 (6.0)	31.4 (6.1)	t = 1.453

	n (%)	n (%)	n (%)	
Education, > high school	66 (47.5)	17 (54.8)	49 (45.4)	X^2^ = 0.866
Employment status, not working	52 (37.1)	13 (41.9)	39 (49.8)	X^2^ = 0.392
Homeless past 3 months, yes	27 (19.4)	13 (43.3)	14 (12.8)	X^2^ = 13.973**
Sexual behaviors, past 3 months				
No. of sex partners, > 1	68 (48.6)	21 (67.7)	47 (43.1)	X^2^ = 5.858*
Unprotected anal intercourse (UAI), yes	80 (57.1)	21 (67.7)	59 (54.1)	X^2^ = 1.827
Transactional sex,^b^ yes	12 (8.6)	7 (22.6)	5 (4.6)	X^2^ = 9.971*
Substance Use Behaviors, past 3 months				
Injection drug use (IDU), yes	9 (6.4)	5 (16.1)	4 (3.7)	Fisher’s exact = 0.025*
Substance use (non-IDU), yes	28 (20.0)	7 (22.6)	21 (19.3)	X^2^ = 0.166
Mental Health, past 3 months	Mean (SD)	Mean (SD)	Mean (SD)	
Psychological distress^c^	4.8 (3.6)	7.3 (3.5)	4.1 (3.4)	t = − 4.689**

^a^Enacted stigma—defined as 3-items each measuring whether the participant reported having experienced (yes, no) verbal harassment, physical violence, and/or discrimination in the past 3 months; the items were summed and any value > 1 was classified as enacted stigma compared to no report of enacted stigma

^b^Transactional sex—defined as a self-report (yes, no) of giving or receiving sex in exchange for money, drugs, or something else in the past 3 months

^c^Psychological distress—defined by a 5-item scale with each item measured on a 4-point frequency scale ranging from never, once or twice, a few times, to many times; the items were summed to create one cumulative ordinal scale

^d^*p-value ≤ 0.05; **p-value ≤ 0.001

In bivariate analysis, enacted sexual minority stigma was significantly associated with homelessness (X^2^ = 3.973; p < 0.001), > 1 sex partner (X^2^ = 5.858; p = 0.016), transactional sex (X^2^ = 9.971; p = 0.002), IDU (Fisher’s exact = 0.025; p = 0.025) and psychological distress (t = − 4.689; p < 0.001) in the past 3 months (Table 1). In addition to these findings, number of sex partners was significantly associated with homelessness (X^2^ = 4.223; p = 0.040), UAI (X^2^ = 9.760; p = 0.002), and psychological distress (t = − 2.158; p = 0.033) in the past 3 months (Table 2). Transactional sex was significantly associated with homelessness (X^2^ = 12.704; p < 0.001), IDU (Fisher’s exact = 0.003; p = 0.003), non-IDU substance use (X^2^ = 7.383; p = 0.007) and psychological distress (t = -3.868; p < 0.001) in the past 3 months. IDU was significantly associated with homelessness (Fisher’s Exact = 0.014; p = 0.014) and transactional sex (Fisher’s Exact = 0.003; p = 0.003) in the past 3 months.

**Table 2:** Participant characteristics by three outcomes in the past 3 months—number of sex partners, transactional sex,^a^ and injection drug use (IDU) – associated with enacted stigma^b^ among Black gay and bisexual men (MSM) participating in the 9-month study visit of the USHINE study, Baltimore City, 2019–2020 (N = 140)

Characteristics	Overall N = 140	No. sex Partners (> 1) n = 68 (48.6)	No. sex Partners (0–1) n = 72 (51.4)	Test statistic^d^	Transactional sex^a^ n = 12 (8.6)	No transactional sex^a^ n = 128 (91.4)	Test statistic^d^	IDU n = 9 (6.4)	Non-IDU n = 131 (93.6)	Test statistic^d^
	Mean (SD)	Mean (SD)	Mean (SD)		Mean (SD)	Mean (SD)		Mean (SD)	Mean (SD)	
Demographics										
Age, years, mean (SD)	31.0 (6.1)	30.0 (6.3)	31.9 (5.8)	T = 1.865	32.1 (6.8)	30.9 (6.0)	t = -0.640	32.8 (5.8)	30.9 (6.1)	t = -0.903

	*n (%)*	*n (%)*	*n (%)*		*n (%)*	*n (%)*		*n (%)*	*n (%)*	
Education, > high school	66 (47.5)	32 (47.1)	34 (47.9)	X^2^ = 0.010	5 (41.7)	61 (48.0)	X^2^ = 0.178	3 (33.3)	63 (48.5)	Fisher’s exact = 0.498
Employment status, not working	52 (37.1)	23 (33.8)	29 (40.3)	X^2^ = 0.624	6 (50.0)	46 (35.9)	X^2^ = 0.929	3 (33.3)	49 (37.0)	Fisher’s exact = 1.000
Homeless past 3 months, yes	27 (19.4)	18 (26.5)	9 (12.7)	X^2^ = 4.223*	7 (58.3)	20 (15.8)	X^2^ = 12.704**	5 (55.6)	22 (16.9)	Fisher’s exact = 0.014*
Sexual behavior, past 3 months										
No. of Sex Partners, > 1	68 (48.6)	~	~	~	7 (58.3)	61 (47.7)	X^2^ = 0.501	6 (66.7)	62 (47.3)	Fisher’s exact = 0.316
Unprotected anal intercourse (UAI), yes	80 (57.1)	48 (70.6)	32 (44.4)	X^2^ = 9.760*	7 (58.3)	73 (57.0)	X^2^ = 0.008	6 (66.7)	74 (56.5)	Fisher’s exact = 0.732
Transactional sex,^a^ yes	12 (8.6)	7 (10.3)	5 (6.9)	X^2^ = 0.501	~	~	~	4 (44.4)	8 (6.1)	Fisher’s exact = 0.003*
Substance use behaviors, past 3 months										
Injection drug use (IDU), yes	9 (6.4)	6 (8.8)	3 (4.2)	Fisher’s exact = 0.316	4 (33.3)	5 (3.9)	Fisher’s exact = 0.003*	~	~	~
Substance use (non-IDU), yes	28 (20.0)	17 (25.0)	11 (15.3)	X^2^ = 2.066	6 (50.0)	22 (17.2)	X^2^ = 7.383*	3 (33.3)	25 (19.1)	Fisher’s exact = 0.383
Mental health, past 3 months										
Psychological distress,^c^ mean (SD)	4.8 (3.6)	5.5 (3.4)	4.2 (3.7)	T = -2.158*	8.5 (4.7)	4.5 (3.3)	t = -3.868**	6.1 (2.8)	4.7 (3.7)	t = -1.112

^a^Transactional sex—defined as a self-report (yes, no) of giving or receiving sex in exchange for money, drugs, or something else in the past 3 months

^b^Enacted stigma—defined as 3-items each measuring whether the participant reported having experienced (yes, no) verbal harassment, physical violence, and/or discrimination in the past 3 months; the items were summed and any value > 1 was classified as enacted stigma compared to no report of enacted stigma

^c^Psychological distress—defined by a 5-item scale with each item measured on a 4-point frequency scale ranging from never, once or twice, a few times, to many times; the items were summed to create one cumulative ordinal scale

^d^*p-value ≤ 0.05; **p-value ≤ 0.001

In unadjusted logistic regression, report of experience of enacted sexual minority stigma significantly increased the odds of reporting in the past 3 months: > 1 sex partner [odds ratio (OR) 2.77, 95% confidence interval (CI) (1.19, 6.44)], transactional sex (OR 6.07, 95% CI 1.77, 20.77) and IDU (OR 5.05, 95% CI 1.27, 20.13) (Table 3, Series A). After adjusting for age and education, the significant associations remained. Report of experience of enacted sexual minority stigma significantly increased the odds of reporting in the past 3 months: > 1 sex partner [adjusted OR (aOR) 2.59, 95% CI 1.10, 6.08], transactional sex (aOR 6.94, 95% CI 1.95, 24.73) and IDU (aOR 6.11, 95% CI 1.46, 25.66) (Table 3, Series B). After additionally adjusting for homelessness, the association between enacted sexual minority stigma and transactional sex remained significant (aOR 4.36, 95% CI 1.11, 17.15), while enacted sexual minority stigma was no longer significantly associated with > 1 sex partner (aOR 2.41, 95% CI 0.96, 6.01) or IDU (aOR 3.76, 95% CI 0.80, 17.78) (Table 3, Series C). Psychological distress was then included in a final model adjusting for age, education and homelessness. Report of experience of enacted sexual minority stigma while still elevated was no longer significantly associated with an increased odds of transactional sex (aOR 3.55, 95% CI 0.78, 16.10) and psychological distress was associated with an increased odds of transactional sex (aOR 5.68, 95% CI 1.27, 25.37) (Table 3, Series D).

**Table 3:** Enacted stigma^a^ in the past 3 months and the unadjusted and adjusted odds (OR, aOR) and 95% confidence interval (CI)^b^ of three outcomes in the past 3 months—number of sex partners, transactional sex^c^ and injection drug use (IDU)—as well as the association with psychological distress^d^ among Black gay and bisexual men (MSM) participating in the nine-month study visit of the USHINE study using logistic regression, Baltimore City, MD, 2019–2020 (N = 140)

	Model 1 No. sex partners (> 1)	Model 2 Transactional sex^c^	Model 3 IDU
Series A	OR (95% CI)	OR (95% CI)	OR (95% CI)
Enacted stigma^a^	**2.77 (1.19, 6.44)**	**6.07 (1.77, 20.77)**	**5.05 (1.27, 20.13)**
Series B	aOR (95% CI)	aOR (95% CI)	aOR (95% CI)
Enacted stigma^a^	**2.59 (1.10, 6.08)**	**6.94 (1.95, 24.73)**	**6.11 (1.46, 25.66)**
Series C			
Enacted stigma^a^	2.41 (0.96, 6.01)	**4.36 (1.11, 17.15)**	3.76 (0.80, 17.78)
Homeless past 3 months, yes	1.91 (0.74, 4.97)	**5.61 (1.40, 22.41)**	**5.00 (1.04, 24.02)**
Series D			
Enacted stigma^a^	–	2.09 (0.45, 9.79)	–
Psychological distress^d^	–	**1.27 (1.04, 1.55)**	–
Homeless past 3 months, yes	–	**5.68 (1.27, 25.37)**	–

^a^Enacted stigma—defined as 3-items each measuring whether the participant reported having experienced (yes, no) verbal harassment, physical violence, and/or discrimination in the past 3 months; the items were summed and any value > 1 was classified as enacted stigma compared to no report of enacted stigma

^b^Statistical significance was defined as a 95% CI that did not cross 1.0, are indicated in bold, and adjusted models were adjusted for age and education

^c^Transactional sex—defined as a self-report (yes, no) of giving or receiving sex in exchange for money, drugs, or something else in the past 3 months

^d^Psychological distress—defined by a 5-item scale with each item measured on a 4-point frequency scale ranging from never, once or twice, a few times, to many times; the items were summed to create one cumulative ordinal scale

## Discussion

In this analysis among Black MSM from a longitudinal cohort study in one mid-Atlantic city, experiences of enacted sexual minority stigma because of sexual orientation were pervasive with more than one fifth of the group reporting one or more experiences of enacted sexual minority stigma including experiences of verbal harassment, physical violence, and discrimination. The current findings were similar to findings among an urban cohort of MSM in 2014 which found that 18% of men reported verbal abuse, and 4% reported physical assault [28], and much higher than a study where 4% reported experiences of external homophobic discrimination among a largely white MSM study in the U.S. recruited from Facebook [29].

The men in the current cohort also had high rates of sexual and drug risk behaviors—almost one in two reported more than one sex partner, nearly one in ten reported transactional sex and 6% reported IDU in the past 3 months. Compared to the NHBS conducted in 23 cities in 2017, the report of transactional sex in this study is similar and report of IDU in our study is nearly triple the proportion reported by Black MSM in the NHBS for a longer time period (past 12 months) [30]. In addition, almost 40% reported high levels of psychological distress in our study, which is almost one in two men, and is higher than the 32% scoring high on psychological distress in a study among MSM attending clinics in one city in Australia from 2008 to 2009 [31].

Those who experienced enacted sexual minority stigma because of their sexual orientation had a threefold, almost sevenfold and sixfold increased odds of higher numbers of sex partners, engagement in transactional sex and report of IDU in the past 3 months, respectively, independent of age and education. After accounting for homelessness, the association between enacted sexual minority stigma and transactional sex remained, and after including psychological distress, the association was diminished and no longer significant. These results suggest a positive and significant association between enacted sexual minority stigma and multiple sex and drug risk behaviors. While not directly comparable to our study, a meta-analysis including studies from 1992 to 2017 showed that Black MSM (compared to White and Latinos) who experienced homophobia were at significantly increased odds of reporting any sexual risk behavior (OR 1.55, 95% CI 1.28, 1.88) [15]. Black MSM bear a disproportionate burden of HIV and syphilis diagnoses and engagement in sexual and drug risk behaviors such as transactional sex increases the risk. Other research has looked at pathways from experiences of homophobia to other sexual and drug risk behaviors among MSM and found significant associations between these experiences of homophobia, inconsistent condom use, and increased HIV/STI risk [32].

These findings are similar to previous work among young Black MSM which demonstrated a positive association between experiences of stigma and psychological distress, even in the presence of social support, a factor that previously was found to be protective [21]. Other types of stigma may be mitigated by social support (i.e., internalized stigma), but enacted sexual minority stigma specifically may lead to psychological distress independent of known protective factors.

### Limitations and Strengths

This analysis has a number of limitations. The study population of Black MSM at the 9-month study visit was smaller than we would have liked and meant that the number of those reporting some risk behaviors such as transactional sex and injection drug use was small. This resulted in wide confidence intervals and may have limited our power to detect significant differences. Given this constraint, additional work is needed to further test this relationship in a larger study population. Our analyses, however, suggest that the 9-month analytic sample was similar to Black MSM not included from baseline. Another limitation is that there is the potential for reverse causality to explain in part the association between enacted sexual minority stigma and sexual and drug risk behaviors. The study’s cross-sectional design with the key measures of enacted sexual minority stigma and psychological distress only measured at 9-months limited our ability to determine the temporality of the relationships of interest. It is possible that, for example, engagement in transactional sex leads to enacted sexual minority stigma exposure. Additionally, measurement of transactional sex was limited in this analysis and did not include information on meeting venues, type of sex work, or the role that income played in engagement in sex work. Also, this analysis largely portrays the activity of transactional sex as an STI/HIV behavioral risk which may underappreciate any benefits of transactional sex (i.e. financial and human connection benefits) [33]. This analysis is also limited by exploring only a one measure of stigma, enacted sexual minority stigma against their sexual orientation, when there may be multiple experiences of different types of stigma among these men. Additionally, the data is reliant on self-report measures of stigmatized behavior, which may be subject to reporting bias. This may be minimal in this study because surveys were self-administered using ACASI and in a confidential setting. Lastly, we aimed to address potential issues of confounding with our a priori framework and exploratory analysis results, which informed which covariates to adjust for, but acknowledge that residual confounding may still be an issue.

This analysis addresses some key gaps in the literature. Previous work has looked at the relationship between enacted sexual minority stigma and various high-risk sexual behaviors, but few have looked at the specific relationship between enacted sexual minority stigma and transactional sex among urban Black MSM. It may be that the experience of enacted sexual minority stigma, psychological distress, and transactional sex is unique among Black MSM and may help to explain racial disparities in HIV and syphilis rates. In addition, participants in this sample were recruited from multiples sites including clinic- and non-clinic-based settings, which in contrast to other studies, may improve the generalizability of the findings to other similar urban settings.

## Conclusions

These results highlight some of the crucial psycho-social components that may be driving engagement in high-risk sexual and drug risk behaviors among Black MSM and placing them at risk for HIV and syphilis acquisition and transmission. These findings support advocacy for intersectional approaches to understand and address experiences of stigma among Black MSM given their dual minority (racial and sexual) status, and their high rates of HIV in the U.S. [34]. These findings also support the importance of interventions to reduce enacted sexual minority stigma in local communities as this may reduce psychological distress and mitigate the HIV and syphilis disparities among urban Black MSM.

## Data Availability

Not applicable.
